# Building Resilient Care Systems for a Globally Aging Society: Insights From Nursing Research

**DOI:** 10.1097/jnr.0000000000000714

**Published:** 2025-11-26

**Authors:** Heng-Hsin Tung

**Affiliations:** National Yang Ming Chiao Tung University, Department of Nursing

Population aging and healthcare workforce shortages together pose a critical challenge to societies across the globe. (Boniol et al., 2022; World Health Organization [WHO], 2025). By 2030, one in six people worldwide will be over the age of 60 and millions of healthcare providers will be needed to meet the increased demand for related services (WHO, 2025). This tension between greater care needs and limited human resources compels us to rethink how health systems can support both patients and providers while fostering resilience.

This issue of *The Journal of Nursing Research* features important studies that shed light on the psychosocial, emotional, and professional dynamics currently shaping healthcare in aging societies. These articles illustrate the loneliness and vulnerability experienced by older adults and the emotional strains, workforce instability, and challenges of sustaining personal accomplishment experienced by nurses and nurse practitioners. They also highlight the promise of resilience and volunteerism as key assets in building stronger care systems.

One of the articles, on loneliness in older adults receiving hemodialysis, reveals that the generally moderate level of loneliness experienced in this population is strongly linked to fatigue and low social support. Nurses are uniquely positioned to address the psychosocial dimensions of care, particularly among older patients who rely on life-sustaining treatments. Equally significant are the findings on psychological distress in nurse practitioners in the aftermath of the COVID-19 pandemic. Stress was found to be highest among practitioners over 35 years and in those with fewer than 10 years of experience, revealing the enduring psychological toll of infectious disease crises. This underscores the necessity for tailored organizational support that considers age, family responsibilities, and career stage. In addition, the included study on emergency department nurses demonstrates the interplay between emotional exhaustion, support, and turnover intention. Emotional support was found to mitigate the effect of exhaustion on intention to leave in nurses, pointing to concrete strategies using peer networks, mentorship, and well-being programs that may directly strengthen workforce retention. The theme of resilience emerges clearly in one of the included studies that explores well-being and resilience in nurse practitioners, with findings revealing personal accomplishment to be strongly influenced by resilience factors such as competence, high standards, and the ability to embrace change. These insights highlight the importance of fostering environments that not only retain practitioners but also help them flourish professionally (Burau et al., 2022). Finally, an article on older adult volunteers demonstrates the value of engaging aging populations as active contributors to community care. While older volunteers bring experience and dedication, their needs for training and support vary by educational background, age, and experience. Thus, structured assessments and ongoing training are essential to maximizing their contributions and promoting active aging.

Together, these studies present a coherent message: addressing global aging and workforce shortages requires attention to both patients and providers that is supported by resilient systems and engaged communities. Nursing research continues to illuminate solutions that are evidence-based, compassionate, and sustainable.
